# Clinical features and prognostic factors of pediatric Langerhans cell histiocytosis: a single-center retrospective study

**DOI:** 10.3389/fmed.2024.1452003

**Published:** 2025-01-15

**Authors:** Yunfeng Lu, Liying Liu, Qi Wang, Bingju Liu, Ping Zhao, Guotao Guan, Yunpeng Dai

**Affiliations:** Department of Pediatric Hematology and Oncology, Shandong Provincial Hospital Affiliated to Shandong First Medical University, Jinan, China

**Keywords:** Langerhans cell histiocytosis, chemotherapy, pediatric oncology, *BRAF*^V600E^ inhibitor, prognosis

## Abstract

**Purpose:**

To retrospectively evaluate the clinical features and prognostic factors of pediatric LCH patients treated in a single center of China.

**Methods:**

Pediatric LCH cases were treated following the SD-LCH protocol at the Affiliated Provincial Hospital of Shandong First Medical University in Jinan, China. An analysis was conducted on 82 recently identified LCH cases to retrospectively evaluate the initial symptoms, therapeutic alternatives, and extended results. Follow-ups were conducted until July 31, 2023.

**Results:**

The median age at diagnosis was 2 (0.25–12) years. 42 (51.2%) were SS-LCH, and 40 (48.8%) were MS-LCH. The most common organ involved was bone (82.9%). Over the 16-year follow-up period, the 5-year EFS and OS rates were 75.2 ± 5% and 90.9 ± 3.3%, respectively. The cumulative reactivation rate was 23.2%. The 5-year EFS rate in SS-LCH and MS-LCH patients were 90.2 ± 4.6% and 58.8 ± 8.3%, and the 5-year OS rate in SS-LCH and MS-LCH patients were 90.2 ± 4.6% and 81.2 ± 6.5%, respectively. The 5-year OS and EFS rate in RO+ LCH and RO− LCH patients were 79.5 ± 7.5%, 53.8 ± 9.6% and 87.5 ± 11.7%, 76.2 ± 14.8%, insignificantly. Multivariate Cox regression showed that liver involvement predicted poor EFS and hematological system involvement was an independent prognostic factor for OS. Detection of the *BRAF*^V600E^ mutation and targeted therapy significantly improved the prognosis post-2017.

**Conclusion:**

Liver or hematological system involvement indicates a poor prognosis, and the SD-LCH protocol improves prognosis for pediatric LCH patients.

## 1 Introduction

Langerhans cell histiocytosis (LCH) is a unique neoplastic disorder brought on by abnormal myeloid differentiation. Its clinical manifestations are widely varied and range from solitary benign lesions to aggressive multisystem involvement. The reported LCH prevalence is 2.6–8.9 incidences per 1 million children aged ≤14 years of age (1). This study aims to elucidate the clinical characteristics, treatment modalities, and prognostic factors of LCH in children aged 14 years and below, providing valuable insights for optimizing therapeutic strategies.

The diagnosis of LCH is primarily based on histopathological examination, with immunohistochemical staining for CD1a and/or CD207 (Langerin) being crucial for confirmation; the presence of Birbeck granules on electron microscopy serves as a definitive diagnostic marker (2). Additionally, *BRAF*^V600E^ mutation is the most common molecular genetic alteration, especially in MS-LCH. Previous studies have highlighted the significance of the *BRAF*^V600E^ mutation in LCH, which is present in approximately 50–60% of cases and is associated with a more aggressive disease course (3). The constrained sample size of this study resulted in a *BRAF*^V600E^ mutation-positive rate of 38.2%. Furthermore, the analysis revealed no significant association between *BRAF*^V600E^ mutations and resistance to first-line treatment, the impact of this mutation on treatment response and long-term outcomes remains to be fully elucidated.

Current treatment protocols for LCH vary depending on the extent of disease involvement. Single-system LCH (SS-LCH) often requires localized therapy, whereas multisystem LCH (MS-LCH) necessitates systemic treatment. Among the current treatment regimens are DAL-HX83/90, JLSG-96/02, and LCH-I to LCH-IV protocols, but optimal therapy has been elusive. In 2010, recurrent *BRAF*^V600E^ mutations were initially reported in LCH samples, suggesting that *BRAF*^V600E^ and MAPK network targeting interventions can be used to treat LCH (3). Despite these efforts, relapse rates remain high, and the management of recurrent disease poses significant challenges. Targeted therapies have shown promise in recent studies, offering new avenues for treatment.

The prognosis of LCH is influenced by several factors, including the extent of organ involvement, age at diagnosis, and response to initial therapy. While overall survival (OS) rates have improved with advances in treatment, event-free survival (EFS) remains suboptimal, particularly in patients with multisystem disease. Identifying reliable prognostic markers and optimizing therapeutic regimens are critical for improving long-term outcomes in these patients.

In this study, we retrospectively analyzed data from children diagnosed with LCH in our center over a 16-year period. We aimed to characterize the clinical features, treatment approaches, and prognostic factors associated with LCH in this population. By examining a large cohort of pediatric patients, we sought to provide a comprehensive overview of the disease and identify potential areas for therapeutic intervention. Our findings underscore the importance of early diagnosis, tailored treatment strategies, and ongoing research into targeted therapies for improving the prognosis of children with LCH.

## 2 Materials and methods

### 2.1 Participants

Pediatric LCH patients aged ≤14 years who needed systemic treatment after preliminary evaluation were recruited for analysis between March 2007 and March 2023 at the Affiliated Provincial Hospital of Shandong First Medical University in Jinan, China. The corresponding pathological diagnoses were obtained from multiple tertiary hospitals in China, and histological findings corroborated with LCH diagnosis, as per the International Lymphoma and Leukemia Classification in 2009 criteria. A definitive LCH diagnosis was made via a positive CD1a and/or CD207 (Langerin) staining within cells from the lesion site, as evidenced by immunohistochemical staining (positive for S100 and CD1a or CD207) or via Birbeck granule identification under electron microscopy within the same cells. Patient follow-up information was recorded until July 31, 2023. We received informed written consent from all study participants and obtained ethical approval from the review board of the participating institution (SWYX: NO.2024–051). This investigation strictly followed the 1964 Declaration of Helsinki’s ethical standards and subsequent revisions.

### 2.2 Data acquisition and organ involvement

We retrospectively extracted the following patient information from electronic medical records: patient demographics, disease traits, treatment modalities, EFS, and OS. Our primary endpoints for patient and disease traits included symptoms, patient age, sex, complete blood count, liver function, presence of masses, and organomegaly (namely, hepatomegaly and splenomegaly). Lastly, radiological data was also acquired from thoracic CT, abdominal ultrasound, cerebral MRI with or without vertebral MRI, and radionuclide bone scans. *BRAF*^V600E^ mutation was identified via qPCR or ddPCR. Next, we categorized patients into two cohorts based on their disease severity and organ involvement: SS-LCH and MS-LCH. The SS-LCH cohort was defined as harboring lesions within a single organ or system, namely, the bone (single or multiple bones), skin, lymph nodes, hypothalamic–pituitary/central nervous system, and other organs (ex. thyroid, thymus). This cohort included two sub-cohorts: SS-s LCH (one lesion within a single system or organ) and SS-m LCH (multiple lesions within a single system) (4). The MS-LCH cohort was defined as harboring lesions in >1 organ or system, and it included two sub-cohorts, namely, Risk Organ (RO; the liver, spleen, and hematological system) positive (RO+) LCH and RO− LCH. RO involvement represented liver enlargement (>3 cm below the costal margin) and/or hyperbilirubinemia and/or augmented circulating GGT and ALP concentrations; splenic enlargement (>2 cm below the costal margin); and hematological system involvement, evidenced with anemia (hemoglobin <100 g/L, infants <90 g/L, not due to other causes), and/or leukopenia (white blood cell count <4.0 × 10^9^/L), and/or thrombocytopenia (platelets <100 × 10^9^/L), with or without biopsy-proven bone marrow infiltration (5). Progressive disease was defined as the progression of signs or symptoms and/or the appearance of new lesions. Relapse/reactivation was defined as the reappearance of signs and symptoms after either complete disease resolution or after a period of disease control that persisted for >3 months on maintenance therapy.

### 2.3 Intervention

Systemic therapy was prescribed for the following patients: SS-LCH with CNS risk lesions (lesions in particular cranial bone locations, namely, orbital, temporal/mastoid, sphenoid, zygomatic, ethmoid bones, maxilla, sinuses, or anterior/middle cranial fossa excluding vault lesions), SS-LCH with multifocal bone lesions (MFB), SS-LCH with lesions in specific locations (namely, odontoid pegs and vertebral lesions with or without intraspinal soft tissue extension), multisystem LCH (MS-LCH) with or without RO involvement, and cases where the SS-LCH disease state was considered to be progressive.

**Figure 1:**
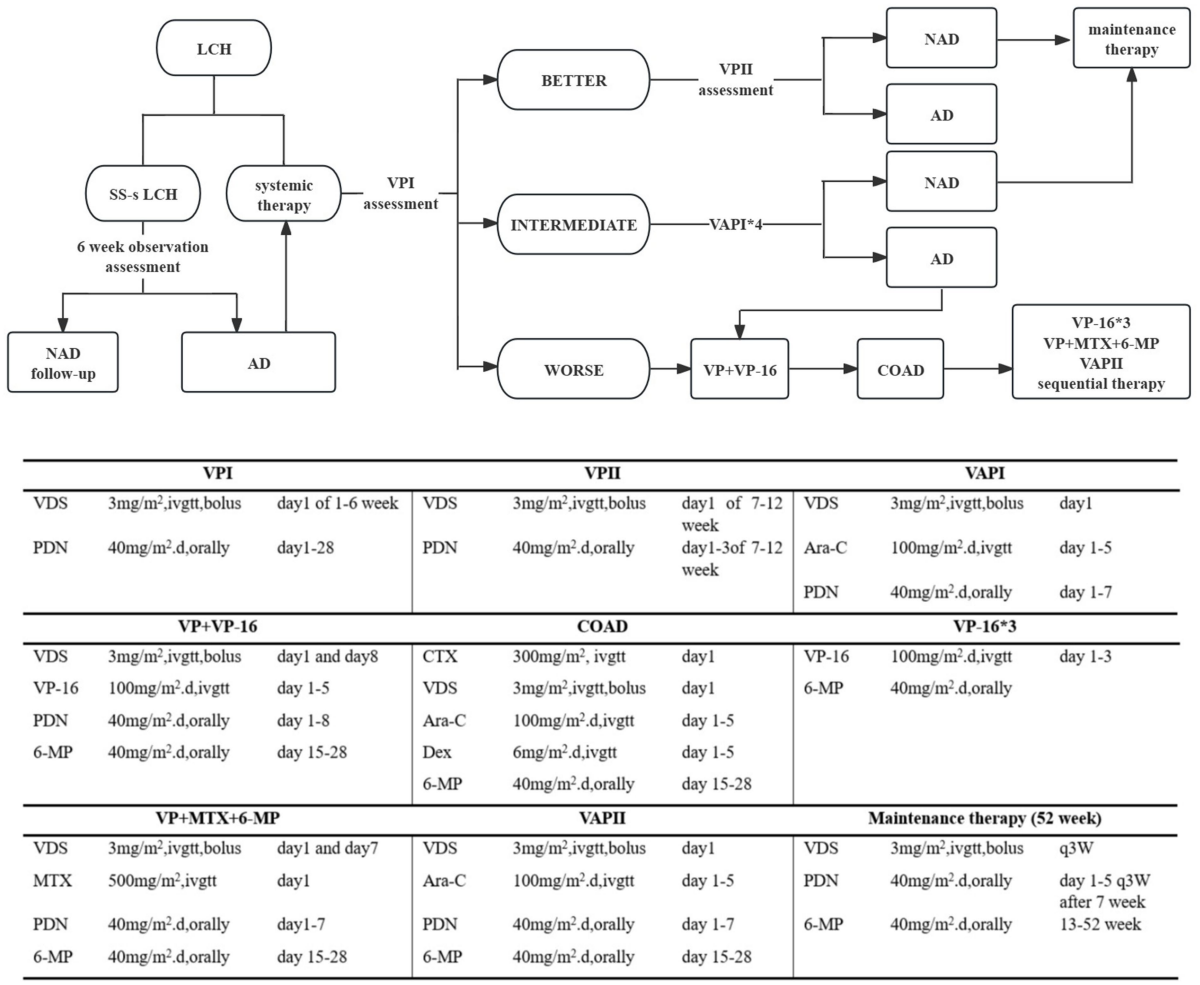
Treatment of LCH patients. NAD, NON-ACTIVE DISEASE no evidence of disease. AD, ACTIVE DISEASE Regression of signs or symptoms, no new lesions; persistence of signs or symptoms, no new lesions; progression of signs or symptoms and/or appearance of new lesions.

Herein, the employed systematic therapy was SD-LCH protocol, and our protocol included administration of vindesine (VDS), prednisone (PDN), 6-mercaptopurine (6-MP), cytarabine (Ara-C), etoposide (VP-16), cyclophosphamide (CTX), and methotrexate (MTX), either alone or in combination with cyclosporin A (Figure 1). In cases of recurring disease, we treated patients as follows: (1) intervention duration was prolonged to 12–24 months; (2) a recurrent protocol involving teniposide, prednisone, vindesine, and cyclosporine A was put in place; (3) cases where PCR testing of pathological tissue, blood, or bone marrow samples confirmed *BRAF*^V600E^ mutation, a dabrafenib-targeted intervention was adopted. All approaches were undertaken with the full consent of the patients’ guardians.

### 2.4 Outcomes

The OS was defined as the duration between initial LCH diagnosis and patient death. In case of survivors, OS was measured based on the last evaluation date. EFS represented the duration between the LCH intervention starting date and onset of the first significant event. Cases without documented events were accounted for by using the last contact date. A significant event was defined as disease reactivation during or after treatment or mortality from any underlying cause.

### 2.5 Statistical analysis

We employed descriptive statistics to examine patient clinical and demographical variables. All data analyses were conducted via the Statistical Package for the Social Sciences for Windows, software version 27.0 (SPSS Inc., Chicago, IL, United States). The examined indexes were evaluated using Mann–Whitney *U* test, *χ*^2^ test or Fisher’s exact test. OS and EFS were predicted via Kaplan–Meier survival analysis and analyzed via log-rank test. Uni- and multivariate Cox regression models were adopted to screen for stand-alone prognostic variables associated with EFS and OS. Variables achieving *p* < 0.05 in univariate analysis were entered into multivariate models. Hazard ratios (HRs) and corresponding 95% confidence intervals (CIs) for OS- and EFS-related factors were predicted via the Cox regression model. Significance was set at *p*-value <0.05.

## 3 Results

### 3.1 Clinical characteristics

We analyzed 82 patients, among which 53 were male (64.6%), producing a male-to-female ratio of 1.8:1. Diagnosis was made at a median age of 2 years and ranged between 3 months to 12 years. Patient follow-up was completed over a median duration of 86.5 months, ranging between 1 and 196 months. Table 1 lists detailed demographics and clinical information of all analyzed patients.

**Table 1:** Clinical characteristics of pediatric Langerhans cell histiocytosis samples (*n* = 82).

Characteristics	*n*	(%)
Sex		
Male	53	64.60%
Female	29	35.40%
Median age at diagnosis, years	2 (0.25–12)	
Clinical classification		
SS-s LCH	13	15.90%
SS-m LCH	29	35.40%
RO+ MS-LCH	31	37.80%
RO− MS-LCH	9	10.90%
Involvement		
Bone	68	82.90%
Eye-ear-oral	37	45.10%
Skin	30	36.60%
Lung	21	25.60%
Liver	20	24.40%
Hematological system	15	18.30%
Spleen	14	17.10%
Lymph nodes	12	14.60%
Pituitary gland	4	4.90%
Central nervous system (CNS)	3	3.70%
Lacrimal gland	1	1.20%
Thyroid	1	1.20%
Thymus gland	1	1.20%
Gastrointestinal tract	1	1.20%
Median follow-up, months	86.5(1–196)	
Progression	10	12.20%
Recurrence	10	12.20%
Abandon treatment	5	6.10%
Death	2	2.44%
Recurrent time, months	29.5(9–149)	
Year of diagnosis		
2007–2017^*^	51	62.20%
2017^*^-2023	31	37.80%

SS-s, single-system disease; SS-m, single-system multifocal disease; MS, multisystem disease; RO, risk organ; CNS, central nervous system. ^*^October 2017.

**Figure 2:**
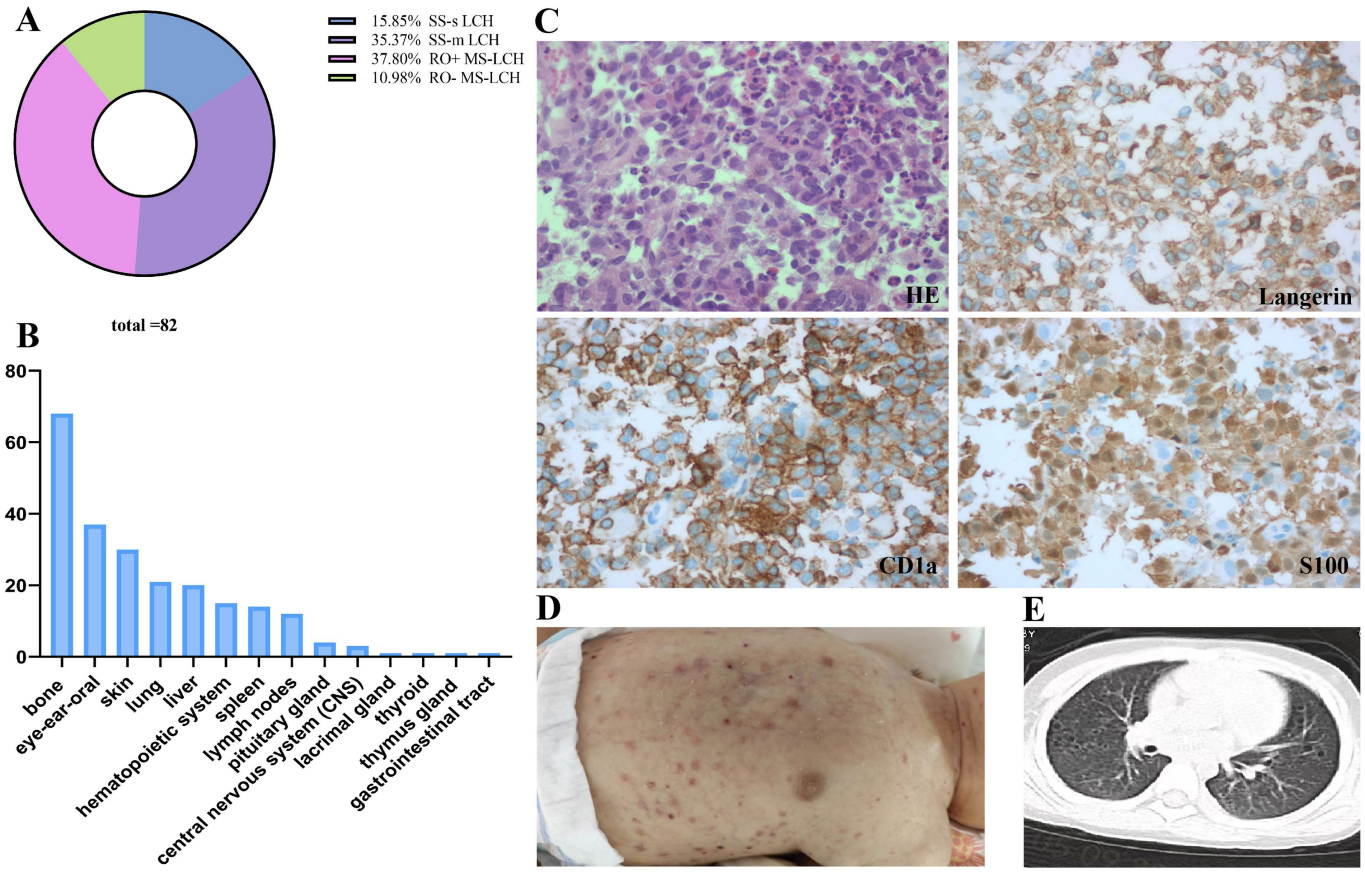
Distribution and manifestations of LCH. **(A)** Distribution of the LCH patients. **(B)** Organ involvement in MS-LCH. **(C)** Histology characteristics of LCH lesion obtained from a puncture biopsy of dorsal mass with pathologic histiocytes and inflammatory infiltrate: hematoxylin and eosin stain demonstrate histiocytes with pale cytoplasm and reniform nuclei, and immunohistochemistry is strongly positive for langerin/CD207, CD1a, and S100a. **(D)** Multiple and polymorphic rash in an LCH infant: cradle cap, purpura, and refractory seborrheic eczema presenting as reddish-brownish crusted papules, petechiae, vesicles, pustules, or painful ulcerations in skinfolds. **(E)** Multiple pulmonary cysts from an MS-LCH patient: High-resolution CT shows a pattern of bilateral reticulonodular, cystic changes and bullae formation, with apical and midlung predominance, sparing the bases and costophrenic angles. SS-s, single-system disease; SS-m, single-system multifocal disease; MS, multisystem disease; RO, risk organ.

Thirteen LCH patients were designated as SS-s LCH (15.9%), 29 as SS-m LCH (35.4%), 31 as RO+ MS-LCH (37.8%), and 9 as RO− MS-LCH (10.9%). Among SS-s LCH patients, 11 displayed singular bone lesions, and 2 revealed distinct, solitary manifestations (1 cutaneous and 1 oral soft tissue). Among SS-m LCH patients, 28 exhibited polyostotic bone involvement, with 1 patient displaying a combination of mucocutaneous lesions (Figure 2A). Among MS-LCH patients, a median of 4 organs was involved, with ranges between 2 and 7. Among the frequently affected organs was the bone (82.9% of cases), then eye-ear-oral involvement (45.1%), skin (36.6%), lung (25.6%), liver (24.4%), hematological system (18.3%), spleen (17.1%), lymph nodes (14.6%), pituitary gland (4.9%), central nervous system (CNS) (3.7%), lacrimal gland (1.2%), thyroid (1.2%), thymus gland (1.2%), and gastrointestinal tract (1.2%) (Figure 2B). Among cases of bone involvement, 49 involved skull bones, and 22 involved specific locations within bones. Histology characteristics and clinical features of LCH lesion were presented in Figures 2C–E.

Among all examined patients, we evaluated the presence of *BRAF*^V600E^ mutations in 48 samples from 34 patients since October 2017. The aforementioned samples were derived from biopsy tissues, plasma, and bone marrow specimens, and *BRAF*^V600E^ mutation was detected via qPCR or ddPCR (Supplementary methods). We detected *BRAF*^V600E^ mutation in 16 total samples from 13 patients (38.2%), with 11 (61.1%) found within biopsy tissue samples, 4 (21.1%) within peripheral blood samples, and 1 (9.1%) within a bone marrow sample; 5 (38.5%) with risk organ involvement, and 5 (38.5%) with progression and recurrence. The presence or absence of the *BRAF*^V600E^ mutation is intricately linked to disease severity among LCH patients (Table 2). We discovered *BRAF* ^V600E^ mutation in 6 (37.5%) MS-LCH patients and in 7 (38.9%) SS-LCH patients (*p* = 0.934).

**Table 2:** Clinical and BRAF^V600E^ mutation in Langerhans cell histiocytosis.

ID	Sex	Age (years)	Disease classification	Involved organ	BRAF^V600E^ mutation	EFS (months)	Disease status	Dasatinib	Risk organ involved
9	M	4	SS-m	B	BT+	149	Recurrence	N	N
39	F	0.25	MS	S, Li, Lu, Bi	BT−	5	Die	N	Y
46	F	1	MS	S, Li, Sp, H	BT+	89	Stable	N	Y
47	M	1	MS	S, Lu, B, LN, Ea	BM−	1.5	Progression	N	Y
50	F	0.42	MS	S, Lu, Li, Sp, H, B	PB−	1	Attrition	N	Y
52	M	1.5	SS-m	B	PB−	1.5	Progression	N	N
53	M	0.92	MS	S, B, LN, H	PB−	69	Stable	N	Y
54	F	1.83	MS	S, Lu, Li, Sp, H	PB−	2	Attrition	N	Y
55	M	7	SS-m	B	BM−	57	Stable	N	N
56	M	10	SS-m	B	BM−	56	Stable	N	N
57	M	1.1	MS	B, S, Ea, Li	BM−	1.5	Progression	N	Y
58	F	7	SS-s	B	BT + BM−	53	Stable	N	N
59	F	1	MS	H, Li, Sp, S, B, Ey	BM−	1	Attrition	N	Y
60	M	6	SS-m	B	BM−	51	Stable	N	N
61	F	1.42	SS-m	S, G	BM−	51	Stable	N	N
62	M	0.92	MS	S, B, H, Lu, Li, Sp	BT+	51	Stable	Y	Y
63	M	0.33	MS	Lu, B, G, S, BM, Li, Sp	BM+	3	Progression	Y	Y
64	M	1.5	SS-m	B	PB−	49	Stable	N	N
65	M	12	SS-m	B	BM−	48	Stable	N	N
66	M	2	SS-m	B	BT−	50	Stable	N	N
67	M	1.5	MS	Lu, B, S	PB−	45	Stable	N	Y
69	F	2	SS-m	So, LN	PB+	36	Stable	Y	N
70	M	5	SS-m	B	BT−	28	Stable	N	N
71	M	0.75	MS	S, Lu, Th, LN, B, Ea, H	BT + PB+	26	Stable	Y	Y
72	F	9	SS-m	B	BT+	25	Stable	N	N
73	F	3	MS	B, Lu, LN	BT−	21	Stable	N	Y
74	M	1.5	MS	B, C, Ea	BT + PB+	20	Stable	Y	N
76	M	1.92	MS	Lu, So, Ty, LN, G	BT−	19	Stable	N	Y
77	F	6	SS-m	B, LN, So	BT + PB−	5	Stable	N	N
78	M	8	SS-s	B	BT + PB−	9	Stable	N	N
79	M	1.92	MS	B, P, Li, Sp, S, LN, LG	BT + PB+	5	Stable	Y	Y
80	M	2	SS-m	B	BT−	4	Stable	N	N
81	M	3	SS-s	B	BT + PB−	15	Stable	N	N
82	M	3	SS-m	B	BT−	9	Stable	N	N

M, male; F, female; MS, multiple system; SS-s, single system single lesion; SS-m, single system multiple lesions; P, pituitary; Bi, bile duct; Lu, lung; B, bone; Li, liver; S, skin; LN, lymph nodes; Ty, thyroid; Sp, spleen; G, gingiva; LG, lacrimal gland; Ey, eyelid; Ea, ear; Th, thymus; C, central nervous system; So, soft tissue; H, hematopoiesis; PB, peripheral blood; BM, bone marrow; BT, biopsy tissue; N, no; Y, yes.

### 3.2 Intervention and patient outcome

**Figure 3:**
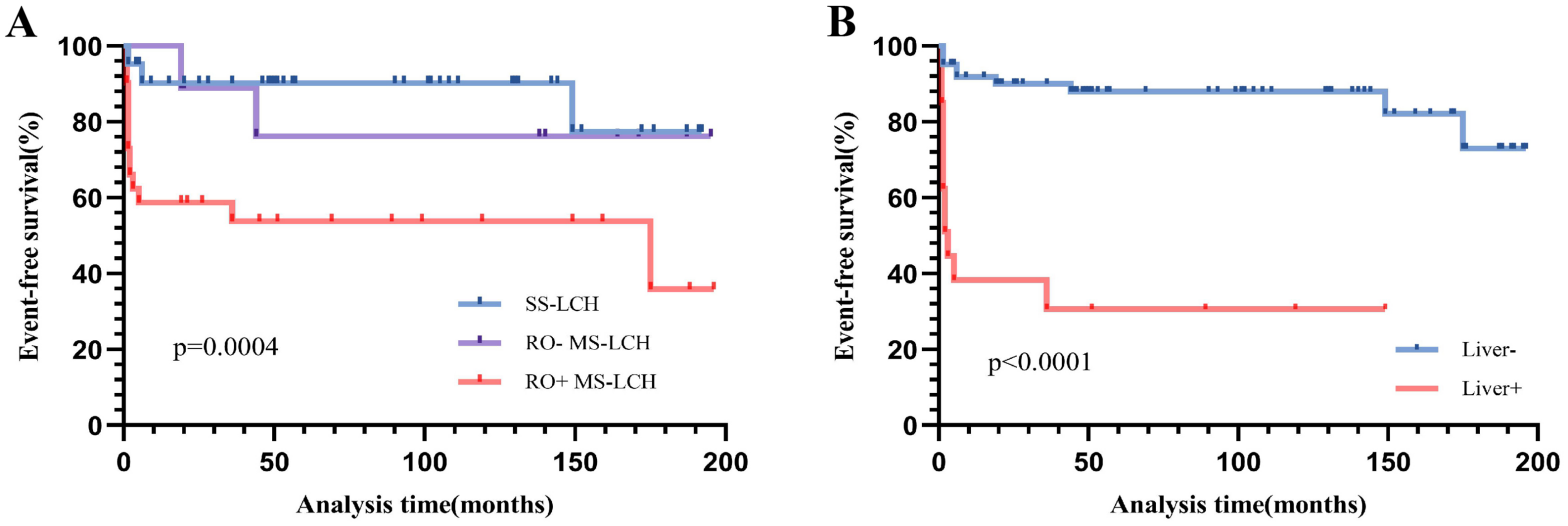
Event-free survival (EFS) for SS-LCH, RO− MS-LCH and RO+ MS-LCH **(A)**, EFS according to liver involvement **(B)**.

**Figure 4:**
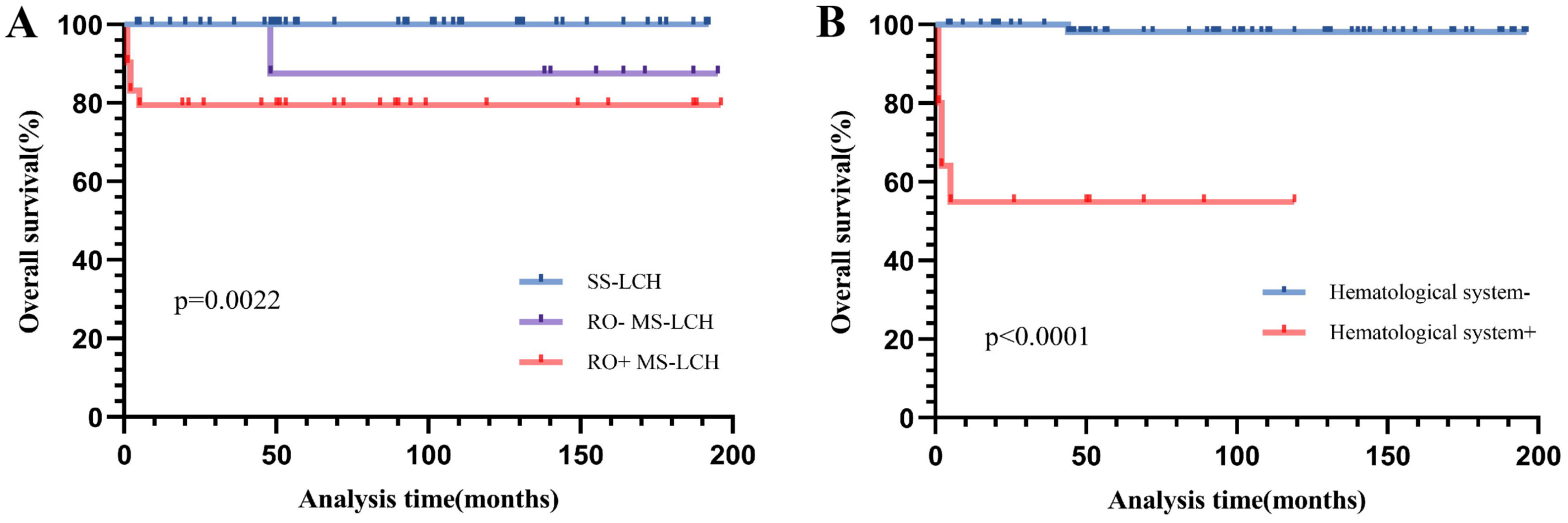
Overall survival (OS) for SS-LCH, RO− MS-LCH and RO+ MS-LCH **(A)**, OS according to hematological system involvement **(B)**.

Among all participants, 2 expired during treatment, 5 died post-treatment, and 3 additional patients were lost to follow-up. The remaining 72 patients were survivors. Over a 16-year follow-up period, the predicted 5-year EFS rate was 75.2% ± 5%, and the OS rate was 90.9% ± 3.3%. The Disease Control Rate (DCR) was 65.9%, and the cumulative reactivation rate was 23.2%. Of note, incidences of secondary tumors were not observed. Our comparison of the SS-LCH and MS-LCH cohorts revealed a 5-year EFS rate of 90.2% ± 4.6 and 58.8% ± 8.3%, respectively (*χ*^2^ = 8.793, *p* = 0.003). Similarly, the 5-year OS rates were 90.2% ± 4.6 and 81.2% ± 6.5% (*χ*^2^ = 8.202, *p* = 0.004), respectively. Additionally, *BRAF*^V600E^ mutation, observed since October 2017, elicited a marked influence on patient outcomes. The 5-year EFS rate before October 2017 was 68.1% ± 6.6%, and it rose to 89.9% ± 5.5% post-October 2017 (*χ*^2^ = 3.889, *p* = 0.049). Likewise, the 5-year OS rate before October 2017 was 86.1% ± 4.9%, and the percentage rose to 100% after October 2017 (*χ*^2^ = 4.11, *p* = 0.043). Please refer to Figures 3, 4 for the EFS and OS for SS-LCH, RO− MS-LCH, and RO+ MS-LCH following SD-LCH-based protocol. The EFS and OS of lung involvement were added in Supplementary Figure S1.

Next, we assessed stand-alone prognostic factors for LCH patients using multivariate Cox regression analysis. Our EFS-based prognostic variables included the involvement of the liver, spleen, hematological system/RO, skin, and bone, as well as patient age and clinical stratification. Based on our analysis, liver involvement (HR 14.795, 95% CI 2.675–81.834) was a strong prognostic indicator of poor EFS (Table 3 and Figure 3). In univariate analysis, which examined common OS prognostic variables, the patient’s age at diagnosis, gender, as well as involvement of the liver, spleen, hematological system/RO, skin, and bone showed remarkable association with the 5-year OS rate. Taken together, the aforementioned variables are strong influences of the overall survival outcomes of LCH patients (Table 3). However, based on our multivariate Cox analysis results, only hematological system involvement (HR 22.939, 95% CI 2.46–213.879) was a stand-alone risk factor for OS among LCH patients (Table 3 and Figure 4).

**Table 3:** Univariate and multivariate Cox models for event-free survival (EFS) and overall survival (OS).

	Univariate Cox		Multivariate Cox	
	*p*-value	HR (95% CI)	*p*-value	HR (95% CI)
Event-free survival (EFS)				
Age	0.013	0.678 (0.499–0.92)		
SS/MS	0.007	4.005 (1.462–10.969)		
RO involvement	0.001	4.541 (1.824–11.307)		
Bone	0.009	0.296 (0.119–0.735)		
Soft tissue	0.032	0.37 (0.149–0.918)		
Liver	<0.001	8.932 (3.555–22.439)	0.002	14.795 (2.675–81.834)
Spleen	0.001	4.658 (1.833–11.834)		
Hematological system	<0.001	5.362 (2.13–13.493)		
Skin	0.012	3.03 (1.272–7.214)		
Overall survival (OS)				
Gender	0.048	5.252 (1.017–27.132)		
Age	0.012	0.15 (0.034–0.654)		
RO involvement	0.024	11.576 (1.39–96.392)		
Bone	0.001	0.067 (0.013–0.348)		
Liver	0.003	23.811 (2.852–198.828)		
Spleen	0.004	9.109 (2.008–41.334)		
Hematological system	0.001	40.582 (4.814–342.102)	0.006	22.939 (2.46–213.879)
Skin	0.022	11.991 (1.441–99.786)		
Skull bones	0.036	0.103 (0.012–0.86)		

#### 3.2.1 SS-LCH

Among the 52 SS-LCH patients, 28 (66.7%) were male, producing a 2:1 male-to-female ratio. The median age at diagnosis was 4 years (range, 5 months–12 years), and the 5-year OS rate was 100%. Among the SS-LCH cohort, 13 were noted as SS-s LCH and 29 patients as SS-m LCH. Among the SS-m LCH patients, 4 exhibited disease progression, and 1 experienced disease recurrence. The 5-year EFS rate among SS-s LCH patients was 100%; among SS-m LCH patients, it was 85.9% ± 6.5%. However, there was no significant difference (*χ*^2^ = 2.023, *p* = 0.155).

#### 3.2.2 MS-LCH

Among the 40 MS-LCH patients, 25 were male, producing a 1.7:1 male-to-female ratio. The median age at diagnosis was 1.45 years, ranging between 3 months and 8 years. Among these patient populations, 31 were designated as RO+ MS-LCH, and 9 as RO− MS-LCH. During a median follow-up of 70.5 months (range, 1–196 months), 7 patients died. Among them, 1 patient with RO− MS-LCH died of severe sepsis during chemotherapy after relapse, 1 patient with RO+ MS-LCH died of disease progression, and 5 patients with RO+ MS-LCH died of abandoning treatment. In addition, three patients were lost to follow-up, six patients had disease progression, two patients with RO− MS-LCH, and three patients with RO+ MS-LCH had disease reactivation. The 5-year RO+ LCH patient OS rate was 79.5% ± 7.5%, whereas the same for RO− MS-LCH patients was 87.5% ± 11.7% (*χ*^2^ = 0.502, *p* = 0.479). Statistically, there was no significant difference in OS rates between the two cohorts. In terms of the 5-year EFS rate, RO+ MS-LCH patients were at 53.8% ± 9.6%, and RO− MS-LCH patients at 76.2% ± 14.8%, however, this too did not reach statistical significance (*χ*^2^ = 2.45, *p* = 0.118).

#### 3.2.3 Recurrent/progressive LCH

Over the median 86.5 months follow-up duration (range, 1–196 months), 10 patients reported recurrence, and another 10 experienced disease progression. The integrated progression and recurrence rate was 23.2%. The median recurrence duration was 29.5 months (range, 9–149 months) post-initial diagnosis. Among 10 patients experiencing recurrence, 6 relapsed once, and 4 had recurrences twice. Six had relapsed status before being treated with the SD-LCH protocol, 2 underwent recurrent protocol therapy, and 3 received extended chemotherapy. Nine recurrences survived, but 1 patient expired due to sepsis during the recurrent protocol therapy.

Among 10 patients who showed disease progression, 1 relapsed but achieved remission following extended chemotherapy, 8 benefitted from upgraded intervention, and lastly, 1 patient achieved remission through oral dabrafenib targeted therapy. Table 4 details the aforementioned patient records.

**Table 4:** Clinical characteristics and treatment in Langerhans cell histiocytosis recurrence and progression.

ID	Sex	Age (years)	Disease classification	Risk organ involved	Disease status	SD (Initial/Recurrent treatment, months)	Survival condition
6	F	4	MS	N	R	12	Alive
7	M	2	MS	Y(Lu)	R*	16/12	Alive
9	M	4	SS	N	R*	22/Rec 4	Alive
11	M	6	SS	N	R	14	Alive
17	M	2	MS	N	R	18/Rec 6	Alive
20	M	11	SS	N	R	12	Alive
30	M	2	MS	Y	R*	18/8	Alive
36	M	2	MS	N	R	20/3	Die
31	M	3	MS	Y	R*	13	Alive
33	F	2	SS	N	P and R	23	Alive
14	M	3	SS	N	P	12	Alive
40	F	0.33	MS	Y	P	18	Alive
43	M	3	MS	Y	P	18	Alive
45	F	1.75	SS	N	P	18	Alive
47	M	1	MS	Y(Lu)	P	18	Alive
49	M	8	MS	Y	P	18	Alive
52	M	1.5	SS	N	P	18	Alive
57	M	1.1	MS	Y	P	18	Alive
63	M	0.33	MS	Y	P	18 + Dasatinib	Alive

SD, SD-LCH protocol; Rec, recurrent protocol; R, recurrence; P, progression; *recurrence twice.

## 4 Discussion

Histiocytosis is a rare disorder with abnormal proliferation of histiocytes, affecting various organs, classified by the Histiocytosis Society into five groups in 2016—L, R, C, M, and H—based on clinical and molecular features (4). The key types include LCH, Erdheim-Chester Disease (ECD), Juvenile Xanthogranuloma (JXG), Rosai-Dorfman Disease (RDD), and hemophagocytic lymphohistiocytosis (HLH), each with unique clinical signs and imaging results impacting treatment. LCH symptoms vary widely, commonly presenting as bone pain or fracture, skin papules, soft tissue masses, lymphadenopathy, diabetes insipidus-related polyuria and polydipsia, exophthalmia, and otorrhea, while ECD mainly affects adults with systemic issues and shows multifocal sclerotic lesions on imaging. ECD cells express CD68 (lysosomal macrophage sialic acid protein), CD14 (lipopolysaccharide receptor), CD163 (hemoglobin and haptoglobin clearance receptor), Fascin protein (actin-binding protein) and factor XIIIa (tissue glutaminase), do not express CD1a and langerin, and S100 is rarely positive (6). JXG, the most common NLCH, appears as benign papules in young children but can be aggressive in disseminated cases, with histological features similar to ECD (4, 7). Finally, RDD, common in children and young adults, presents as a large painless neck lymph node with macrophages and may relate to autoimmune, genetic, and malignant diseases (8). Typical histiocytic markers are S100, fascin, CD4, CD11c, CD14, CD68, CD163, even BCL-1 and OCT2 (9). Nevertheless, these four types share a common pathophysiology related to the clonal accumulation of mononuclear phagocytes associated with histiocytosis exhibit mutations in genes such as *CSF1R, ALK, RET, NTRK, RAS, RAF,* or *MAP2K*, which connect the binding of myeloid cell-specific growth factor receptors to the activation of the ERK signaling pathway (10). In LCH or ECD patients, the *BRAF^V600E^* mutation was identified in 50–60% of cases, followed by the *MAP2K* mutation. In JXG patients, *WT*, *MAP2K1*, and *CSF1R* mutations were mainly detected, while RDD patients primarily exhibited mutations in *WT, KRAS, TNFRSF,* and *SLC29A3* (2). Further research is warranted to elucidate their underlying mechanisms and improve therapeutic strategies.

Despite advancements in understanding LCH, challenges remain in its diagnosis and management to avoid irreversible organ damage resulting from late-stage LCH. This complexity necessitates a thorough understanding of its clinical, radiological, and pathological features for accurate diagnosis and effective management. In MS-LCH, the skeletal system is primarily affected, with common lesions found in the skull, vertebrae, and long bones such as the femur and humerus. Radiographs reveal osteolytic lesions characterized by a “punched-out” appearance. MRI and CT scans help detail bone involvement and associated soft tissue masses, which can mimic other conditions such as osteomyelitis, Ewing’s sarcoma, and primary bone tumors (11). Eye-ear-oral involvement accounted for 37% of cases in our study, with symptoms like orbital masses, otorrhea, and gum swelling. In addition, infants may be misdiagnosed with eczema until imaging and histological examinations reveal the appearance of symptoms of liver, spleen, bone, and hematological system involvement. In pulmonary LCH, CT scanning can show cystic lesions, necessitating differentiation from other lung diseases. When evaluating a patient with suspected CNS LCH, it is essential to consider a broad differential diagnosis that includes other histiocytic disorders and neoplastic conditions. For instance, ECD often presents multiple tumorous lesions, predominantly affecting the meninges and vascular structures, whereas LCH lesions are typically solitary and localized to the hypothalamic–pituitary axis (12). Furthermore, endocrine disorders such as central diabetes insipidus are common in LCH and can mimic symptoms seen in other conditions. Histological examination and genetic testing for mutations like *BRAF^V600E^* can provide additional diagnostic clarity associated with LCH (13). Therefore, a multidisciplinary approach, including clinical evaluation, imaging, and molecular analysis, is essential for accurate diagnosis (14).

Furthermore, race was found to be a factor in the incidence of LCH, with Hispanics having a higher risk (15). The incidence of LCH among Asians is primarily based on regional epidemiological statistics. Herein, we present one of China’s largest and longest single-institutional retrospective studies on LCH children. All pediatric participants underwent systemic therapy, highlighting their disease’s severity. A detailed comparison between MS-LCH and SS-LCH patients revealed that the median diagnosis age for MS-LCH patients (1.45 years, range: 0.25–8 years) was significantly younger than SS-LCH patients (4 years, 0.42–12 years, Z = −5.014, *p* < 0.001). The age distribution of onset is similar to that of developed countries (16). Recent research suggested that LCH cells might arise from bone marrow-derived progenitors (17), and Getlinks’ study of the crucial effects of ERK activation in LCH cells on hematopoietic progenitors, including mutated genes related to RAS⁃RAF⁃MEK⁃ERK signaling pathway (18), especially MS-LCH. We speculate that this may be a reason for the younger onset of MS-LCH. Of note, more male versus female LCH patients suffered from severe LCH. As in our findings, bone is the most susceptible organ to be involved, but it suggests a good prognosis (19), and lung involvement has no impact on OS and EFS rates. Notably, the presence of the *BRAF*^V600E^ mutation was identified in a substantial subset of patients, which has implications for targeted therapy. The study also demonstrated that the SD-LCH protocol, which includes a combination of various drugs, effectively managed both SS-LCH and MS-LCH cases. Furthermore, the use of dabrafenib in *BRAF*^V600E^-positive recurrent cases showed promising results. These findings align with our initial hypothesis that a comprehensive treatment approach tailored to the severity and genetic profile of the disease can improve clinical outcomes in pediatric LCH patients.

The LCH-I study showed that etoposide and vinblastine were equivalent in probability of survival and toxicity for the early treatment of LCH. Also, previous studies have found that cases of LCH treated with etoposide have a risk of secondary leukemia of approximately 1/100 (20). In our study, in addition to one recurrent case that expired due to severe sepsis, more side effects in children were mild to moderate (World Health Organization score I–II). Although methotrexate was eliminated from the LCH-IV protocol, an adults’ study showed that methotrexate effectively treated LCH patients (21). Due to the limited sample size employed in our study, whether MS-LCH with skin involvement would benefit from treatment with low-dose methotrexate remained to be investigated. The development of neurodegeneration was not monitored during follow-up. One reason might be that Neurodegeneration did not manifest clinically for more than 10 years after the initial diagnosis of LCH (22) or due to the effective use of cytarabine and MARK pathway targeting therapy (23). Hence, during chemotherapy and follow-up for LCH patients, recurrence, drug-related toxicity, and long-term complications were all necessary surveillance items.

Since the individuals in the study were all patients who needed systemic treatment after preliminary evaluation, there was a high percentage of MS-LCH patients. During the follow-up of 16 years, the 5-year EFS and OS rates were 75.2 ± 5% and 90.9 ± 3.3%, respectively. Cumulative reactivation rate was 23.2%. The 5-year EFS rate in SS-LCH and MS-LCH patients were 90.2 ± 4.6% and 58.8 ± 8.3% (*χ*^2^ = 8.793, *p* = 0.003), respectively. The 5-year OS rate in SS-LCH and MS-LCH patients were 90.2 ± 4.6% and 81.2 ± 6.5% (*χ*^2^ = 8.202, *p* = 0.004), respectively. Following the International Organization Cell Society LCH-III regimen treatment covering 12 months, RO+ MS-LCH patients reported a 5-year OS rate of 84.0%, with a recurrence rate of 27.0% (24). Employing the JLSG-02 protocol, the Japanese LCH Study Group achieved a 5-year OS rate of 91.7% among RO+ MS-LCH patients, along with a 5-year EFS of 46.2% among RO+ MS-LCH and 69.7% among RO− MS-LCH patients (25). Similarly, using the JPLSG-12 protocol, another group achieved a 3-year EFS of 66.1 and 51.1% among RO− MS-LCH and RO+ MS-LCH patients, respectively (26). Of note, in France, the RO+ MS-LCH OS rate was 91.7%, which was attributed to 2-CdA-Ara-C therapy for refractory cases (27). Moreover, 5-year OS, PFS, and relapse rates for the BCH-LCH 2014 study were 99.2, 54.5, and 29.3%, respectively (28). Adopting a modified LCH-III protocol, JLSG-02 chemotherapy with vemurafenib, one group in China achieved predicted 5-year OS and EFS rates of 98.8 and 74.6%, respectively (29). In this report, our 5-year OS and EFS rates were 90.9 ± 3.3% and 75.2 ± 5%, respectively. In the last 5 years post-October 2017, the OS and EFS rates rose to 100% and 89.9 ± 5.5%, respectively, with a corresponding 5-year OS rate of 81.2% among MS-LCH patients. In particular, the 5-year EFS was 53.8 for RO+ MS-LCH patients and 76.2% for RO− MS-LCH patients. Despite recent advancements, the 5-year cumulative reactivation rate for RO+ MS-LCH patients remained incredibly high at 29%, similar to rates reported in developed nations (24).

According to our findings, most recurrences occurred within 6 months after diagnosis, but three recurrences occurred 20, 44, and 149 months after diagnosis, respectively. The augmented survival rates may be due to prolonged and intense initial intervention spanning 18–24 months, potentially impacting the rapidity and severity of response, particularly among RO+ MS-LCH cases. The ongoing LCH-IV investigation (NCT02205762) aims to elucidate whether intervention prolongation to 24 months and chemotherapy intensification with cytarabine introduction effectively minimize LCH recurrence and reactivation in pediatric patients (1). At present, the optimal second-line intervention for persistent or recurring LCH among RO− MS-LCH patients remain unknown. The current recommendations are to extend treatment to enhance outcomes and minimize relapse. Our findings highlight the significance of continued exploration into the optimal treatment modalities for LCH patients, particularly those with a persistent or relapsing LCH population. There is also a critical need for efficacious second-line therapies. We did not find a statistical difference between RO− MS-LCH patients and RO+ MS-LCH patients. The reason might be that the treatment plan of increasing chemotherapy intensity or prolonging chemotherapy time correspondingly once there was progress according to the results of the evaluation before each course of treatment had improved the prognosis of RO+ MS-LCH patients.

The recent reports of augmented OS and EFS rates among LCH patients are likely due to dabrafenib, a newly designed *BRAF* inhibitor. Mutations within the MAPK axis, particularly within *BRAF* and *MAP2K1*, are found in approximately 80% of LCH patients. Thus, these are regarded as LCH driver mutations (10, 14). The *BRAF*^V600E^ oncogene is known to accelerate cellular senescence and regulate immune escape, which are proposed mechanisms behind LCH pathophysiology (4, 14). Over the years, *BRAF* and *MAP2K1* inhibitors have been commonly used and with good success. However, there is a significant challenge of rapid relapse after treatment discontinuation (4). Thus, additional explorations are necessary to establish the long-term safety of inhibitor therapy.

Emerging evidence revealed that the circulating cell-free *BRAF*^V600E^ (cf*BRAF*^V600E^) mutational analysis of LCH patient plasma is strongly associated with a satisfactory clinical outcome (30). Likewise, herein, we demonstrated that cf*BRAF*^V600E^ concentration monitoring is a robust approach to predicting patient prognosis, determining optimal time for intervention cessation, and identifying disease reactivation. Additional investigations are warranted to determine whether cf*BRAF*^V600E^ analysis can replace *BRAF*^V600E^ mutation detection within tissue samples. Among the benefits of cf*BRAF*^V600E^ mutational analysis is its non-invasive approach and its precise prediction of disease severity and treatment response. Therefore, extensive research into cf*BRAF*^V600E^ application and reliability is key to enhancing LCH treatment and patient outcomes (29).

Bortezomib, an NF-κB inhibitor, diminishes senescence-associated secretory phenotype (SASP) cytokine gene expression, thereby suppressing CD1a + CD207+ LCH cell proliferation. Hence, bortezomib may be a promising new candidate for LCH intervention (31). In a murine LCH model, *PD-1* blockers and *MAPK* inhibitors demonstrated a synergistic impact that abrogated T-cell failure in LCH (32). Diamond et al. reported that *CSF1R* is ubiquitously expressed in LCH and critically modulates LC migration and differentiation. Therefore, *CSF1R* signal blocking is another potential candidate for LCH therapy (33, 34). The aforementioned evidence highlights the complex nature of LCH pathophysiology, demonstrates the significance of *BRAF*^V600E^ in enhancing cellular differentiation, and reveals the therapeutic relevance of targeting major LCH progression-related networks. Bortezomib, *PD-1* blockers, *CSF1R* blockers, or *MAPK* inhibitors are robust modulators of inflammatory responses and abrogate LCH cell proliferation. As such, their role in LCH therapy may be significant. However, additional investigations are warranted to validate the relevance of these drugs in LCH treatment.

The clinical implications of our study on LCH in children under 14 years are profound and multifaceted. Firstly, we demonstrated that liver or hematological system involvement indicates a poor prognosis. Secondly, identifying *BRAF*^V600E^ mutations in a significant subset of patients underscores the potential for targeted therapies, such as dabrafenib, to improve outcomes in this population. Our findings indicate that applying *BRAF*^V600E^ targeted therapy is associated with a better prognosis, as evidenced by the improved 5-year EFS and OS rates post-October 2017 when targeted therapy became more prevalent. This suggests that routine screening for *BRAF*^V600E^ mutations should be integrated into clinical practice to tailor treatment strategies effectively. In addition, the stratification of patients based on disease severity and organ involvement provides a framework for personalized treatment plans. The significant difference in survival rates between these groups highlights the need for more aggressive and prolonged treatment regimens for MS-LCH patients.

It is noteworthy to acknowledge some limitations in this study. Several factors, like the retrospective study design, single-center Chinese sample population, and relatively small patient population, may restrict the generalizability of our findings. Additionally, this study concentrated on the *BRAF*^V600E^ mutation. However, there could be multiple other genes that can potentially affect LCH etiology. Therefore, comprehensive molecular profiling is optimal for LCH screening, treatment selection, and patient prognosis. We also acknowledge the need to identify therapeutic targets beyond *BRAF*^V600E^ and their potential efficacy in LCH management. Long-term follow-up is essential to fully understand the disease’s progression and the efficacy of treatments over time. Future research should focus on prospective randomized controlled clinical trials and larger, longer cohorts to validate these results and refine treatment protocols further. Meanwhile, there is a need for more in-depth studies on the molecular mechanisms underlying LCH to develop more effective and personalized therapeutic strategies.

## Data Availability

The original contributions presented in the study are included in the article/Supplementary material, further inquiries can be directed to the corresponding author.
