# Supplementary figures and images for: Clinical features and prognostic factors of pediatric Langerhans cell histiocytosis: a single-center retrospective study
Source: Front Med (Lausanne). 2025 Jan 15;11:1452003. doi: 10.3389/fmed.2024.1452003 (PMC11774849; doi:10.3389/fmed.2024.1452003)

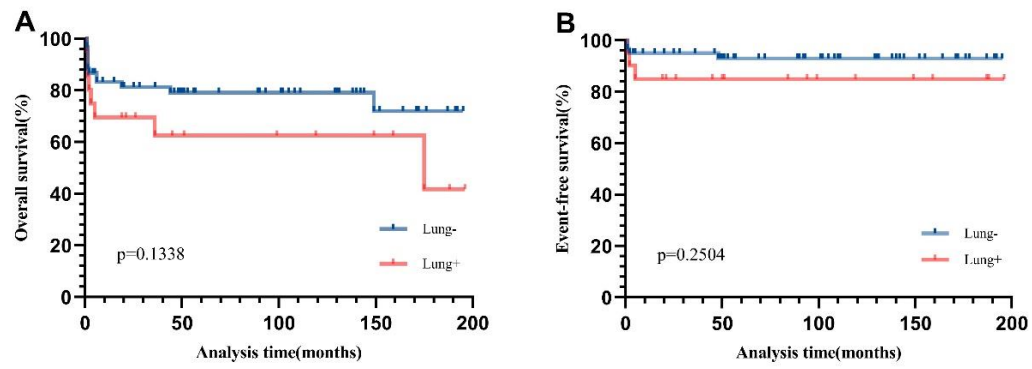

**FIGURE S1 Overall Survival (OS) (A) and Event-Free Survival (EFS) (B) according to lung involvement.**

Supplement: Supplementary file 1 [file Data_Sheet_1.pdf]
