# Supplementary material for: Clinical features and prognostic factors of pediatric Langerhans cell histiocytosis: a single-center retrospective study
Source: Front Med (Lausanne). 2025 Jan 15;11:1452003. doi: 10.3389/fmed.2024.1452003 (PMC11774849; doi:10.3389/fmed.2024.1452003)
Supplement: Supplementary file 2 [file Data_Sheet_2.pdf]

Supplementary Methods

Blood samples were obtained in EDTA-containing tubes from individuals diagnosed with LCH. Plasma cfDNA was extracted utilizing the QIAamp Circulating Nucleic Acid Kit (Qiagen) following the manufacturer's guidelines and preserved at -80°C until analysis. Utilizing the QX200TM Droplet Digital PCR System (Bio-Rad), the existence and quantity of cfBRAF^{V600E} were assessed. As a positive control, Tru-Q7 (1.3% Tier) Reference Standard DNA (Horizon Discovery) was employed, while gDNA from the white blood cells of healthy donors served as the negative control. The assay quantified BRAF^{V600E} mutation fragments in a patient sample relative to wild-type BRAF fragments. Each sample underwent testing in duplicate at a minimum. To establish the detection limit of the ddPCR assay, Tru-Q7 Reference Standard DNA was progressively diluted into healthy donor gDNA to achieve mutant allele percentages ranging from 8% to 0.01%. The detection threshold of the assay was identified as 0.1%.
